# Improvement of a Genetic Transformation System and Preliminary Study on the Function of *LpABCB21* and *LpPILS7* Based on Somatic Embryogenesis in *Lilium pumilum* DC. Fisch

**DOI:** 10.3390/ijms21186784

**Published:** 2020-09-16

**Authors:** Shengli Song, Rui Yan, Chunxia Wang, Jinxia Wang, Hongmei Sun

**Affiliations:** 1 Key Laboratory of Protected Horticulture of Education Ministry and Liaoning Province, College of Horticulture, Shenyang Agricultural University, Shenyang 110866, China; ssl_syau@163.com (S.S.); yanrui2020@sina.cn (R.Y.); 2004500043@syau.edu.cn (C.W.); wangjinxia74@163.com (J.W.); 2 National and Local Joint Engineering Research Center of Northern Horticultural Facilities Design and Application Technology, Shenyang 110866, China

**Keywords:** *Lilium pumilum* DC. Fisch., somatic embryogenesis, auxin transport, genetic transformation, CRISPR/Cas9

## Abstract

Auxin transport mediates the asymmetric distribution of auxin that determines the fate of cell development. *Agrobacterium*-mediated genetic transformation is an important technical means to study gene function. Our previous study showed that the expression levels of *LpABCB21* and *LpPILS7* are significantly up-regulated in the somatic embryogenesis (SE) of *Lilium pumilum* DC. Fisch. (*L. pumilum*), but the functions of both genes remain unclear. Here, the genetic transformation technology previously developed by our team based on the *L.* *pumilum* system was improved, and the genetic transformation efficiency increased by 5.7–13.0%. Use of overexpression and CRISPR/Cas9 technology produced three overexpression and seven mutant lines of *LpABCB21*, and seven overexpression and six mutant lines of *LpPILS7*. Analysis of the differences in somatic embryo induction of transgenic lines confirmed that *LpABCB21* regulates the early formation of the somatic embryo; however, excessive expression level of *LpABCB21* inhibits somatic embryo induction efficiency. *LpPILS7* mainly regulates somatic embryo induction efficiency. This study provides a more efficient method of genetic transformation of *L. pumilum*. *LpABCB21* and *LpPILS7* are confirmed to have important regulatory roles in *L. pumilum* SE thus laying the foundation for subsequent studies of the molecular mechanism of *Lilium* SE.

## 1. Introduction

Auxin plays a critical role in plant development by controlling cell expansion, polarity, and organ patterning [1]. Auxin is mainly synthesized in expanding leaves along with shoot apical meristem and is transported to various parts of the plant [2] by polar auxin transport (PAT) [3]. Three crucial plasma membrane (PM) auxin transporters are involved in the PAT system, PIN-FORMED (PIN) auxin efflux facilitators [4], AUXIN1/LIKE AUX1 (AUX1/LAX) influx carriers [5], and ATP-binding cassette (ABC) transporter B family (ABCB) efflux/influx transporters [6]. PIN-LIKES (PILS) located at the endoplasmic reticulum (ER) has also been shown to be involved in auxin transport [7,8].

The ABCB subfamily is the second largest ABC protein subfamily in plants with 10, 18, 21, and 22 ABCB members in *Physcomitrella patens*, *Selaginella moellendorfii*, *Arabidopsis thaliana*, and *Oryza sativa*, respectively [9]. Some members of the ABCB subfamily have been confirmed to be involved in auxin transport; ABCB1, ABCB4, ABCB14, ABCB19, and ABCB21 are the most intensively studied members [9,10]. ABCBs can regulate auxin transport over long and short distances [11] and plant ABCBs are selective to auxin substrates [12]. For example, ABCB19 [13] and ABCB21 [14] have higher transport activity for IAA (indole-3-acetic acid) than that for NAA (1-naphthaleneacetic acid). ABCB1 [15], ABCB4 [16], and ABCB19 [17] can bind 2,4-D (2,4-dichlorophenoxyacetic acid); however, ABCB4 has a higher affinity for 2,4-D [16]. The AUX1/LAX auxin transport family mainly regulates the influx of auxin [5,18]. However, two members of the ABCB subfamily have different characteristics including ABCB4 and ABCB21, which are auxin concentration-dependent influx/efflux transporter that mediate the influx activity when the cytoplasmic auxin concentration is low and efflux activity when the auxin concentration is high [14,16].

The structure of PILS proteins (PILS1-PILS7) is similar to PIN proteins, although the former constitute an independently evolved protein family [19]. PILS and PIN proteins have only approximately 10–18% sequences similarity; however, both protein families contain auxin transport domains and are involved in intracellular auxin accumulation [7]. PILS protein family may be more ancient and conserved even in unicellular algae [20,21]. PILS proteins regulate intracellular auxin accumulation at the ER and consequent auxin availability for nuclear auxin signaling [7,8,22]. Additionally, non-canonical PIN proteins, such as AtPIN5/6/8, are also located at the ER and participate in intracellular auxin transport [20,23]. The function of PILS proteins in development is unclear, and it is currently believed that PILS proteins can limit the rate of auxin signal transduction and response to external stimuli, such as light and temperature, to regulate auxin-dependent development [8,22]. For example, the light signaling pathway can directly regulate the activity of PILS thus regulating phototropic growth [22]. High temperature can inhibit the expression of PILS6 and increase auxin signal thus promoting organ development [8]. A recent study has demonstrated that PILS proteins are involved in the interaction between brassinosteroid and auxin to regulate plant development [24].

Genetic transformation is an important technical means to study gene function. *Agrobacterium*-mediated transformation is a plant transformation method that employs an efficient and low-cost system that has been widely used for stable gene transfer [25]. In this process, the expression of a tumor-inducing (Ti) plasmid of *Agrobacterium* is necessary for T-DNA (transfer DNA) transfer into the host cells to integrate T-DNA into the host genome [26]; the virulence (*vir*) gene of the TI plasmid determines the transformation ability of *Agrobacterium* [27,28,29]. Activation of the *vir* gene requires signals from the host plants, including phenolic derivatives, acidic pH, monosaccharides, and low PO_4_ [27,28,29,30,31]. Phenols are the major signal components in plants, and phenolic signal can be enhanced by certain sugars, low pH, and low PO_4_ [28,32]. Monocotyledons are generally considered to be insensitive to *Agrobacterium* infection and transformation due to lack of wound response [33] and deficiency in phenolic derivatives [32]. *Lilium* has been one of the recalcitrant monocotyledon species for *Agrobacterium*-mediated transformation until transgenic lily was generated for the first time [34]. Since then, numerous studies on genetic transformation of *Lilium* and several *Lilium* varieties have successfully obtained transgenic plants, such as *Lilium* × *formolongi* [35], *Lilium tenuifolium* oriental × trumpet “Robina” [36], *Lilium* oriental “Sorbonne” [37], and *Lilium cv* “Manissa” [38]. However, due to the strong genotype dependence, low efficiency of stable transformation, poor genetic stability, and difficult regeneration after transformation, this technique is still unable to meet the current requirements of *Lilium* [39]. In 2019, the genetic transformation system of *Lilium pumilum* DC. Fisch. (*L. pumilum*) was successfully established in our study [39]; however, the system is not perfect because it needs a long transformation cycle and its transformation efficiency is relatively lower than that of model plants.

At present, the regulatory role of auxin transport in somatic embryogenesis (SE) remains unclear. Our previous results demonstrated that the expression levels of *LpABCB21* and *LpPILS7* auxin transporters are significantly up-regulated in SE and may play an important regulatory role in *L. pumilum* SE [40]. Moreover, the genetic transformation system of *L. pumilum* developed previously by our team [39] is improved by optimization of the pH of RC (re-suspension solution and co-culture medium), and the germination conditions of resistant calli. In this study, the genetic transformation efficiency is increased after the improvements, and important regulatory roles of *LpABCB21* and *LpPILS7* in *L. pumilum* SE are confirmed.

## 2. Results

### 2.1. Construction of Expression Vectors

**Figure 1:**
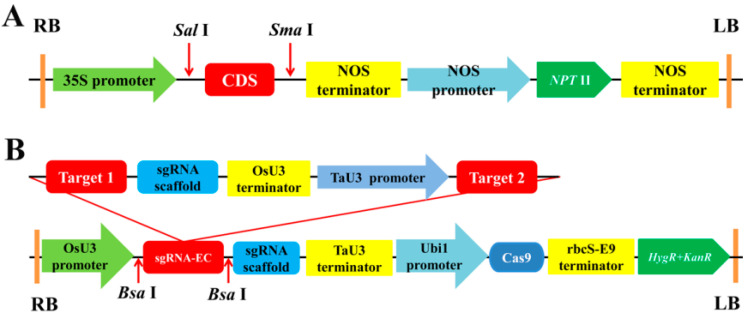
Schematic diagram of the recombinant vector. (**A**) schematic diagram of recombinant vectors OE-*LpABCB21* and OE-*LpPILS7*. (**B**) schematic diagram of recombinant vectors KO-*LpABCB21* and KO-*LpPILS7*. RB and LB are the right and left borders of the recombinant vector, respectively; *NPT* II is a selection marker gene for overexpression lines. *HygR* and *KanR* are selection marker genes for mutant lines. sgRNA-EC is a dual sgRNA expression cassette.

Complete CDSs (coding sequences) of *LpABCB21* (4086 base pairs (bp)) and *LpPILS7* (1461 bp) were inserted into the pRI101-ON vector by double enzymes digestion, and the generated vectors were named OE-*LpABCB21* and OE-*LpPILS7*, respectively (Figure 1A). The sequences information is listed in Supplementary File.

**Figure 2:**
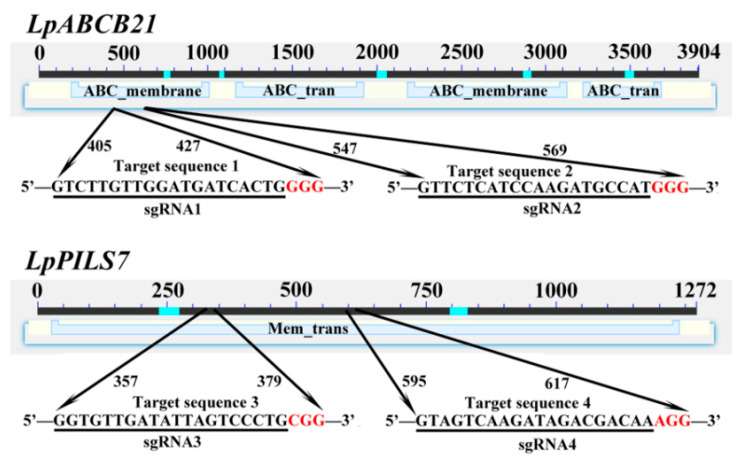
Selection of sgRNA for *LpABCB21* and *LpPILS7* gene editing. The conserved domains and their locations are labeled. The cyan lines correspond to compositionally biased regions and were not used for domain database search. The red letters correspond to the NGG domains.

A 20 bp sgRNA (small-guide RNA) was selected in the conserved domain of the CDS. The sgRNA sites in *LpABCB21* and *LpPILS7* are shown in Figure 2. Dual sgRNA expression cassette was successfully constructed by overlapping PCR (polymerase chain reaction). Dual sgRNA expression cassette was inserted into the pG3H-U3Ub vector using single enzyme digestion, and the corresponding vectors were named KO-*LpABCB21* and KO-*LpPILS7* (Figure 1B).

### 2.2. Comparison of Genetic Transformation Systems before and after the Improvement

**Figure 3:**
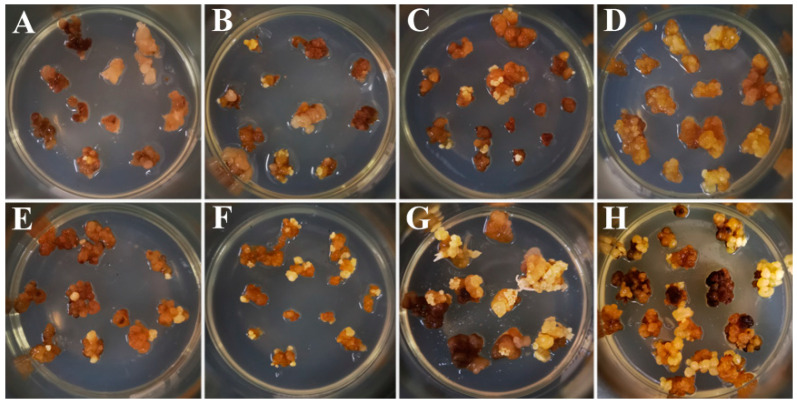
The growth of resistant calli at various pH of RC (re-suspension solution and co-cultivation medium). (**A**–**D**), resistant calli obtained in RC with pH 5.8. (**E**–**H**), resistant calli obtained in RC with pH 5.0. A and E, the results of OE-*LpABCB21* infection. B and F, the results of OE-*LpPILS7* infection. C and G, the results of KO-*LpABCB21* infection. D and H, the results of KO-*LpPILS7* infection.

After two rounds of antibiotic screening, the transformed calli were cultured in somatic embryo induction medium for 30 days. The growth of resistant calli is shown in Figure 3. The results indicate that a higher number of resistant calli masses were generated by the RC5.0 treatment (Re-suspension II and Co-cultivation II) (E–H) than that was generated by the RC5.8 (Re-suspension I and Co-cultivation I) (A–D), and all resistant calli masses had a larger volume and a higher number of clusters.

As shown in Table 1, the number of resistant calli masses obtained by the RC5.0 treatment increased by approximately 2–3-fold.

**Figure 4:**
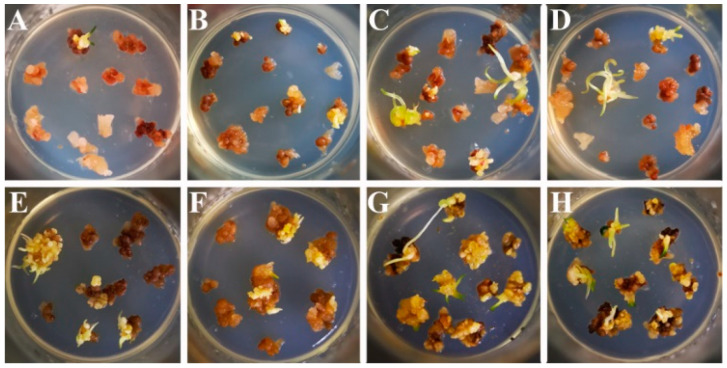
Resistant calli germination. (**A**) and (**E**), OE-*LpABCB21*; (**B**) and (**F**), OE-*LpABCB7*; (**C**) and (**G**), KO-*LpABCB21*; (**D**) and (**H**), KO-*LpPILS7*. (**A**–**D**), Germination I condition; (**E**–**H**), Germination II condition.

As shown in Figure 4, the resistant calli obtained by treatment with RC5.8 and RC5.0 were subcultured for 30 days under Germination I (A–D) and Germination II conditions (E–H), respectively. Thirty-seven OE-*LpABCB21* resistant plants were obtained, including 32 plants under Germination II condition (D) and five plants under Germination I condition (A). Thirty-seven OE-*LpPILS7* resistant plants were obtained, including 30 plants under Germination II condition (F) and seven plants under Germination I condition (B). Fifty-six KO-*LpABCB21* resistant plants were obtained, including 41 plants under Germination II condition (G) and 15 plants under Germination I condition (C), and 63 KO-*LpPILS7* resistant plants, including 52 plants under Germination II condition (H) and 11 plants under Germination I condition (D).

### 2.3. Identification of Transgenic Plants

**Figure 5:**
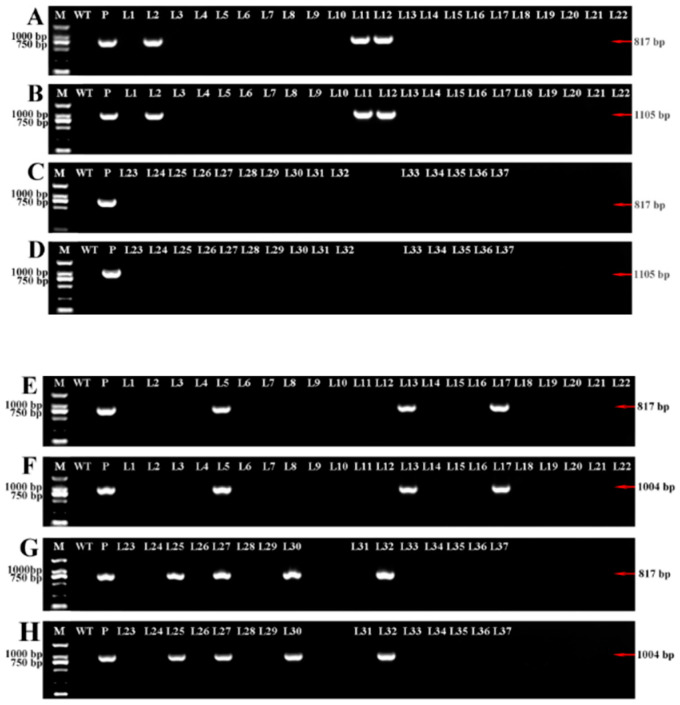
PCR (polymerase chain reaction) identification of OE-*LpABCB21* (**A**–**D**) and OE-*LpPILS7* (**E**–**H**) positive lines. (**A**,**C**,**E**) and (**G**) are the cloning results of *NPT*II gene (817 bp); (**B**) and (**D**) are the cloning results obtained using the primers against the 35S promoter and *LpABCB21* (1105 bp); (**F**) and (**H**) are the cloning results obtained using the primers against the 35S promoter and *LpPILS7* (1004 bp). L33–L37 in (**C**,**D**) and L31-L37 in (**G**,**H**) are obtained before the improvement. The other lines are obtained after the improvement. M, 2 kb DNA marker. WT, wild-type plant. P, vector plasmid control.

As shown in Figure 5, the transgenic plants were identified by PCR. The *NPT*II gene fragment (817 bp) and the specific fragment between the vector and the target gene (1105 bp/1004 bp) were successfully cloned, and the positive plants were identified. After verification, 3 OE-*LpABCB21* positive lines (L2, L11, and L12) and 7 OE-*LpPILS7* positive lines (L5, L13, L17, L25, L27, L30, and L32) were obtained. All lines except L32 were obtained after the improvement.

**Figure 6:**
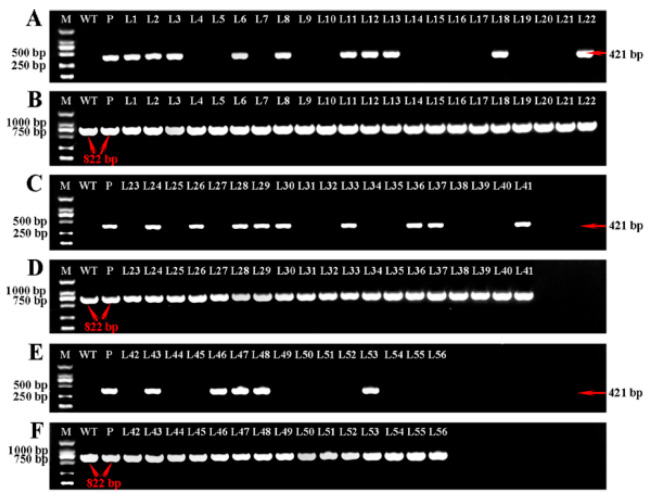

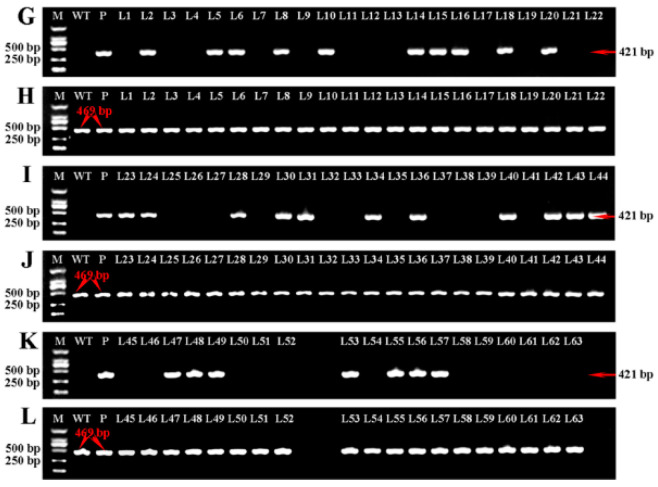
PCR identification of KO-*LpABCB21* (**A**–**F**) and KO-*LpPILS7* (**G**–**L**) positive plants. (**A**,**C**,**E**,**G**,**I**) and (**K**) are the cloning results obtained using the primers against the OsU3 promoter and sgRNA expression cassette (421 bp). (**B**,**D**) and (**F**) are the cloning results of the fragments on *LpABCB21* containing dual sgRNA (822 bp). (**H**,**J**) and (**L**) are the cloning results of the fragments on *LpPILS7* containing dual sgRNA (469 bp). L42–L56 in (**E**,**F**) and L53–L63 in (**K**,**L**) are obtained before the improvement. The other lines are obtained after the improvement. M, 2 kb DNA marker. WT, wild-type plant. P, vector plasmid control.

As shown in Figure 6, the specific fragment (421 bp) between the vector and the sgRNA expression cassette was successfully cloned and the positive plants were identified. After verification, 24 KO-*LpABCB21* positive plants were obtained; however, five plants were obtained before the improvement. 24 KO-*LpPILS7* positive plants were obtained; however, only 5 plants were obtained before the improvement.

**Figure 7:**
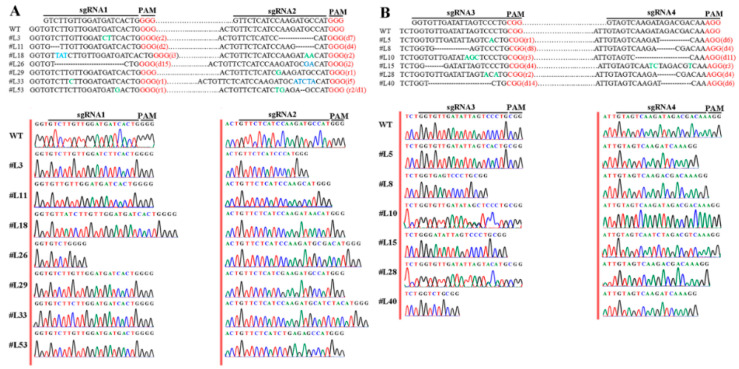
Identification of the mutation modes of KO-*LpABCB21* (**A**) and KO-*LpPILS7* (**B**) mutant lines. r/blue indicate replacements. i/green indicate insertions. d/- indicate deletions. Dots represent sequences between dual sgRNA. PAM represents NGG domains.

To identify the mutation modes of the mutant lines, the cloned PCR fragments from KO-*LpABCB21* and KO-*LpPILS7* positive plants were purified and sequenced; the mutation modes are shown in Figure 7. Mutations occurred in 7 of the 24 KO-*LpABCB21* positive plants, and no mutations occurred in line L29 at the sgRNA1 site. Mutations occurred in six of the 28 KO-*LpPILS7* positive plants, and heterozygous mutations occurred in lines L10 and L28 at the sgRNA3 site.

**Figure 8:**
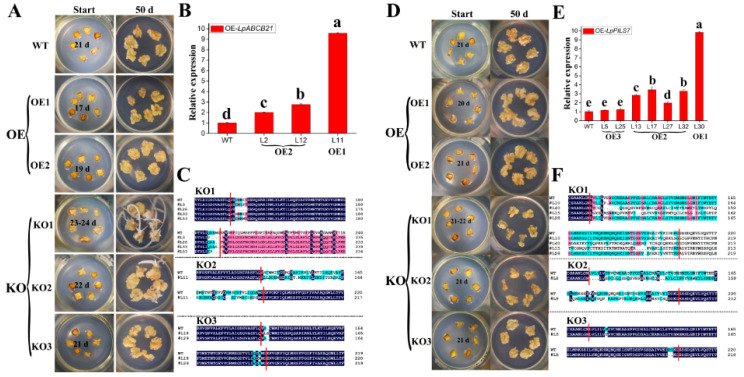
The effects of overexpression and knockout of *LpABCB21* and *LpPILS7* on somatic embryo induction in *L. pumilum*. (**A**), somatic embryo was induced by *LpABCB21* transgenic lines; (**B**), the expression levels of *LpABCB21* in overexpression lines; (**C**), the protein sequence alignment of KO-*LpABCB21* mutant lines; (**D**), somatic embryo was induced by *LpPILS7* transgenic lines; (**E**), the expression levels of *LpPILS7* in overexpression lines; (**F**), the protein sequence alignment of KO-*LpPILS7* mutant lines. WT, wild-type plants; OE, overexpression lines; OE1, the expression level increases by more than 9-fold; OE2, the expression levels increases by 2–4-fold; OE3, the expression level did not change significantly; KO, mutant lines; KO1, frame shift mutations; KO2, mutation between dual sgRNA; KO3, mutations in dual sgRNA. Start, the emergence time of somatic embryo is marked in the image; 50 d, somatic embryo induction for 50 days. Data were analyzed using one-way ANOVA using SPSS; the differences were compared using Fisher’s least significant difference test (LSD) at the 5% significance level. The red vertical bars in C and F represent the edge of dual sgRNA.

Analysis of the changes in the protein sequences after mutations indicated that the protein sequences of KO-*LpABCB21* and KO-*LpPILS7* mutant lines had frame shift mutations, protein mutations between dual sgRNA, and amino acid mutations in dual sgRNA (Figure 8C,F). Three lines had amino acid mutations in dual sgRNA. KO-*LpABCB21* line L18 had insertion of a nonpolar leucine (+L) and replacement of polar asparagine with nonpolar alanine (N→A), and line L29 had replacement of polar negatively charged glutamate with polar glutamine (E→Q). KO-*LpPILS7* mutant line L5 had replacement of polar threonine with nonpolar proline (T→P), and two polar negatively charged aspartic acid residues (-DD) were missing.

Since *LpABCB21* and *LpPILS7* are membrane transporters, the hydrophilicity, hydrophobicity, and transmembrane domains of the mutant protein sequences were estimated by prediction. The results showed that hydrophilicity and hydrophobicity of the proteins were considerably affected by the frame shift mutation and protein mutations between dual sgRNA (B–D, G–J); however, these properties were affected to a lesser extent by the amino acid mutation in dual sgRNA (E, F, K, and L) (Figure S1). The wild-type *LpABCB21* protein sequence consists of 11 transmembrane domains (A). The frame shift mutation resulted in deletion of the third to eleventh transmembrane domains (B, C, D, and E). The protein mutation between dual sgRNA caused deletion of the third transmembrane domain; the distribution of amino acid residues to the inner and outer membrane prior to the missing transmembrane domain was also affected (F). The transmembrane domain of the amino acid mutant with mutation in dual sgRNA was identical to that of the wild-type protein (G and H). The wild-type *LpPILS7* protein sequence consists of 10 transmembrane domains (I). The frame shift mutations resulted in deletion of the third to tenth transmembrane domains (K and M) or the fifth to tenth transmembrane domains (J and L). The protein mutation between dual sgRNA resulted in deletion of the fourth transmembrane domain (N). The transmembrane domain of the amino acid mutant with mutation in dual sgRNA was identical to that of the wild-type protein (O) (Figure S2).

The assay of the expression levels in the overexpression lines showed that the level of *LpABCB21* was significantly increased by 2.0–9.6-fold in 3 OE-*LpABCB21* lines compared with that in wild-type plants (Figure 8B). In 7 OE-*LpPILS7* lines, the expression level of *LpPILS7* was significantly increased by 2.0–9.8-fold compared with that in wild-type plants except lines L5 and L25 (Figure 8E).

### 2.4. Analysis of Genetic Transformation Efficiency

As shown in Table 2, the RC5.0 and Germination II conditions (after improvement) are better than RC5.8 and Germination I conditions (before improvement); the germination rate increased by 17.7–42.1%, the number of resistant plants increased by 2.7–6.4-fold, the number of positive lines increased by 3–6-fold, and the genetic transformation efficiency increased by 5.7–13.0%.

### 2.5. The Effects of LpABCB21 and LpABILS7 on SE

The somatic embryo was formed when the scales of the wild-type plants were cultured in somatic embryo induction medium for 21 days, and the somatic embryo induction rate was 100%. Comparison with somatic embryo induction in the wild-type plants indicated that *LpABCB21* mainly regulated early SE and had a certain influence on regulation of late SE. After overexpression of *LpABCB21*, the emergence time of somatic embryos was advanced by 2–4 days. When the expression level increased by 2.0–2.8-fold, the somatic embryo induction efficiency was not changed. Although the expression levels in line L2 and L12 were significantly different, there were no differences in SE. When the expression level was significantly increased in line L11, somatic embryo induction efficiency decreased by approximately 30–40%, and the emergence time of somatic embryos was advanced by additional 2 days compared with that in line L2 and L12. *LpABCB21* mutation delayed the emergence time of somatic embryos by 1–3 days, and adventitious buds were formed in the late stage. The number of adventitious buds formed by the frame shift mutant (KO1) was the highest, and the adventitious bud induction rate was 79.2% (19/24) (Figure 8A). *LpPILS7* mainly regulated late SE and had little effect on early SE. Only a few lines can advance (OE1: L30) or delay (KO1: L10 and L40) the emergence of somatic embryos by 1 day. Somatic embryo induction efficiency increased by approximately 10–30% when *LpPILS7* expression levels increased by 2.0–9.8-fold, while *LpPILS7* mutations decreased somatic embryo induction efficiency by approximately 10–60% (Figure 8D). Although there were transgenic lines with significantly different expression levels in OE2, there were no significant differences in SE. If the expression level was significant increased by 9.8-fold (OE1), somatic embryo induction efficiency was further increased. The results of somatic embryo induction in various protein mutation modes indicated that proteins with the frame shift mutation (KO1) or the large fragment mutation (KO2) had severely damaged function of the protein; however, the insertion, replacement, and deletion of individual amino acids (KO3) had weaker effect on the function of the protein. For example, *LpABCB21* mutant lines L18 and L29 had almost no effect on SE. The somatic embryo induction efficiency of *LpPILS7* mutant line L5 was reduced by 10%, which was less than the effect of the KO1 and KO2 mutant lines.

## 3. Discussion

The pH of the medium was reported to be crucial for *Agrobacterium* virulence in in vitro tissue culture [41]. The optimal pH for *Agrobacterium* growth is 7.0 [42], but optimal infection of the host occurs at pH 4.8–5.5 [43,44]. In the presence of AS (acetosyringone), the induction of the *vir* gene is favored under the acidic conditions [32], while the expression of the *vir* gene is barely induced at neutral pH; the expression level of the *vir* gene is increased by 1.2–2.0-fold when pH drops from 7.0 to 5.5 [43]. In the co-culture of *Agrobacterium tumefaciens* and plant cells, the expression level of the *vir* gene changes depending on pH and reaches the maximum at pH 5.1 [43]; the expression level of the *vir* gene decreases when pH increases from 5.1 to 5.6 [41]. Genetic transformation efficiency is maximal when pH of the pre-culture or co-culture media is 5.0 [25,45]. The co-culture medium with pH 5.2 is more suitable for the genetic transformation of tomato (*Solanum lycopersicum*) cotyledon [46]. Our study further confirms that low pH can promote *Agrobacterium*-mediated genetic transformation. The number of resistant calli increased when pH of the re-suspension solution and co-culture medium decreased from 5.8 to 5.0 indicating that pH 5.0 is more suitable for the genetic transformation of *L. pumilum* than pH 5.8. However, a pronounced decrease in pH has an inhibitory effect on genetic transformation [35]. Since the genetic transformation efficiency of *L. pumilum* was compared only under the conditions with pH 5.0 and pH 5.8 in this experiment, additional improvement in the genetic transformation efficiency of *Lilium* should consider optimization of pH of the transformation process.

Calcium ions are the major second messenger in plants, and the calcium signaling pathway has been extensively studied [47,48]. Recent studies have shown that CaCl_2_ (calcium chloride) can promote the induction of embryogenic calli (EC) and the germination of somatic embryos. Addition of 10 mM CaCl_2_ increased the germination rate of somatic embryos by 51.79% [49]. The results of the present study indicate that an increase in the content of CaCl_2_ from 0.44 g/L to 1.32 g/L can improve the germination coefficient of somatic embryos. However, the role of CaCl*_2_* in SE is genotype- and variety-dependent [49]. Therefore, additional screening of optimal dosages is necessary for various materials, and the mechanism of CaCl_2_ regulation of the germination of somatic embryo requires additional investigation.

Previous studies have shown that ABCB21 and ABCB4 are bidirectional auxin transport carriers, which control the direction of auxin transport depending on auxin concentration to regulate the auxin level in the cells [14,16]. Recent studies in Arabidopsis have shown that ABCB21 can regulate the transport of auxin in cotyledons, root pericycle, and leaves to regulate normal growth [6]; however, the mechanism of SE regulation by ABCB21 remains unclear. ABCB21 has auxin influx or efflux activities under certain conditions, which is very similar to the pattern observed in ABCB4 [17]. A recent study has shown that ABCB21 initially has auxin uptake activity until intracellular auxin concentration reaches a certain threshold; then, the efflux activity is activated [6]. Our data indicate that various expression levels of *LpABCB21* may also influence auxin transport rate. Several studies have shown that SE is more easily induced when the explants contain high auxin levels [50,51,52], which indirectly indicates that the auxin content in the explants reaches a high level in advance may promote earlier formation of somatic embryos. However, the ratio of auxin to cytokinin determines the development fate of the reproductive organs [53]. These considerations suggest that the content of exogenous auxin or cytokinin entering the explants and the content of endogenous cytokinin and auxin in the explants should be assessed when the exogenous hormone is used to change the original development state of the explants so that the ratio of auxin to cytokinin is always within the threshold range required for specific organ development. In this study, when exogenous auxin was constant, somatic embryos appeared earlier in the overexpression lines indicating that auxin content may increase to a higher level within a certain period of time to reach the auxin threshold required for SE to advance. An increase in the expression of *LpABCB21* may shorten the time required for auxin to reach the threshold of SE to induce an additional advance in the emergence time of somatic embryo. However, if the auxin content is so high that it exceeded a certain threshold, the continued growth of somatic embryo is inhibited. Bielach found that cytokinin application on lateral root primordia delays the appearance of maximum auxin levels and lateral root growth but has no effect on the formation of the lateral root [54]. ABCB21 mutations and cytokinin treatment produce the same results [6]. These studies confirm the results of our experiments. The emergence time of somatic embryos was delayed in the *LpABCB21* mutant lines, and there was no significant influence on the induction efficiency of somatic embryos. It is not clear whether this is the result of functionally redundancy of ABCB4 with ABCB21 during SE or if AUX/LAX1 and ABCB21 coordinate to regulate the exogenous auxin influx.

The function of PILS proteins in plant development is unclear; it is currently believed that PILS proteins can limit the rate of auxin signal transduction and response to external stimuli, such as light and temperature, to regulate auxin-dependent development [8,22]. Our results indicate that the mechanisms of *LpPILS7* and *LpABCB21* involved in auxin regulation may be similar; however, the proteins are associated with different patterns of auxin regulation. The reasons for this difference may be due to two factors. First, PILS protein is located at the ER and controls the accumulation of auxin in the cells. Second, the function of PILS protein mainly involves regulation of auxin signal through binding or releasing auxin and availability of auxin in the nucleus [8,22]; however, ABCB21 is located on the PM and is mainly involved in the balance of extracellular and intracellular levels of auxin. ABCB21 acts by determining the direction of auxin transport based on auxin concentration [14,17]. However, the ultimate result is the same. In in vitro tissue culture, the balance between exogenous and endogenous auxin levels can be adjusted so that the ratio between auxin and cytokinin can be maintained within the threshold range of specific requirements of a developmental process. When *LpPILS7*-overexpressimg and mutant lines induce somatic embryos, the effect on the emergence time of somatic embryos was very small. This phenomenon may be explained by very high rate of auxin transport [6]; hence, the process of exogenous hormone transport into the cells, which is probably determined by *LpABCB21*, is not affected. Our previous study demonstrated that *LpPILS7* expression level is increased in the light culture, and somatic embryo induction efficiency in the light culture is better than that in the dark culture [40]. This experiment confirms that *LpPILS7* can promote *L. pumilum* SE. PILS protein was suggested to bind auxin to limit nucleus auxin signaling [8,22]. PILS6 mutants have an increased auxin signal in the nucleus [8]. The results of this experiment suggest that PILS7 and PILS6 may have the same molecular mechanism of in regulation of auxin signals, which will be verified in the future studies.

In conclusion, this study describes a more efficient method of genetic transformation of *L. pumilum* improved by decreasing the pH of re-suspension solution and co-culture medium to 5.0 and increasing the CaCl_2_ concentration of germination medium to 1.32 g/L; these results are important for subsequent functional genomics studies and molecular breeding in *Lilium*. Our findings confirm that *LpABCB21* and *LpPILS7* have important regulatory roles in *L. pumilum* SE and contribute to determination of the molecular mechanism of *Lilium* SE.

## 4. Materials and Methods

### 4.1. Plant Materials, Strains, and Vectors

*L. pumilum* EC were used as material. EC were obtained according to a previously described method [55]. Cultures were grown at 25 ± 1 °C under a 16/8 h photoperiod with a photosynthetic photon flux density of 36 μmol m^−2^ s^−1^ provided by cool white fluorescent light bulbs (Philips, Shanghai, China).

Plant overexpression vector pRI101-ON was purchased from Takara Biomedical Technology Co., Ltd. (Dalian, China). *Agrobacterium tumefaciens* EHA105 harboring the helper plasmid pVS1-SAH2, CRISPR/Cas9 knockout vector pG3H-U3Ub, and template pCBC-DT1T2.2 were donated by Professor Chen Qijun from China Agricultural University.

### 4.2. Construction of Overexpression and CRISPR/Cas9 Knockout Vectors

Primers with restriction enzyme cutting sites were designed using the Primer 5 software according to the sequences of the target genes and overexpression vectors. The sgRNA was designed according to the PAM/NGG domain on the sequences of the target genes. The sgRNA requires the content of GC% to be higher than 40%, absence of four consecutive thymines, and continuous pairing between sgRNA and target gene less than 8 bp. The primers for overexpression and knockout vector construction are listed in Table S1.

Previously, sequencing verified plasmids of *LpABCB21* and *LpPILS7* were used as templates to clone the CDS of *LpABCB21* and *LpPILS7*. The reaction system volume was 50 μL containing 1 μL of the *LpABCB21*/*LpPILS7* plasmids, 1 μL of *LpABCB21*-F/*LpPILS7*-F (10 μM), 1 μL of *LpABCB21*-R/*LpPILS7*-R (10 μM), 22 μL of ddH_2_O, and 25 μL of PrimeSTAR Max DNA polymerase (Takara, Dalian, China). The incubation was at 98 °C for 1 min; (98 °C 10 sec., 60 °C 5 sec., and 72 °C 1 min) × 30 cycles; and 72 °C for 1 min. The pBCB-MT1T2.2 plasmid was used as a template to clone the sgRNA expression cassettes. The reaction system volume was 50 μL containing 1 μL of the pBCB-MT1T2.2 plasmids; 1 μL of *LpABCB21*-BsF/*LpPILS7*-BsF (10 μM); 1 μL of *LpABCB21*-BsR/*LpPILS7*-BsR (10 μM); 1 μL of *LpABCB21*-F0/*LpPILS7*-F0 (1 μM); 1 μL of *LpABCB21*-R0/ *LpPILS7*-R0 (1 μM); 20 μL of ddH_2_O; and 25 μL of PrimeSTAR Max DNA polymerase. The incubation was at 98 °C for 1 min; (98 °C 10 s, 60 °C 5 s, and 72 °C 30 s) × 30 cycles; and 72 °C for 1 min. After the PCR product was purified, the CDS of the target genes and the sgRNA expression cassettes were inserted into pRI101-ON and pG3H-U3Ub, respectively, by enzymatic ligation.

### 4.3. Improvement of Agrobacterium-Mediated Genetic Transformation System

The genetic transformation method was performed as described previously [40]. In brief, EC were transferred to somatic embryo induction medium for proliferation and subcultured every 30 days until genetic transformation was performed. Light yellowish friable EC were selected as recipient materials. *Agrobacterium* expressing the vectors was grown in a liquid medium until the OD_600_ value reached 0.6. Collected bacteria were subjected to Re-suspension I in the same volume to suspend the bacteria. After addition of 100 µmol AS, the EC that had been precultured in the pre-cultivation medium for 10 days were infected for 15 min. The EC were inoculated into Co-cultivation I for culture for 3 days in the dark, and then inoculated into Selection I and Selection II media for 2 weeks. Finally, transformed EC were inoculated into Germination I for germination. The results of preliminary experiments (Supplementary Table R1 and Figure R1) demonstrated that the germination efficiency of somatic embryos was the highest when non-transformed EC were inoculated in Germination II in the light culture (the light period was 16/8 h). To further improve the genetic transformation efficiency, the following aspects were improved. The original method was used as the control. The media used in the transformation process are shown in Appendix A Table A1.

Improvement of culture condition: The pH of the re-suspended solution and co-culture medium were changed to 5.0. The number of resistant calli was counted.

Improvement of somatic embryo germination condition: EC were inoculated into Germination II for germination. Statistics of somatic embryo germination efficiency was assessed.

### 4.4. Identification of LpABCB21 and LpPILS7 Transgenic Plants

The genomic DNA of the leaves was extracted and used as a template for PCR identification. The primers for identification of transgenic plants are listed in Table S2. Specific primers for identification of overexpression in plants were designed based on the 35S promoter sequence (35S-F), the inserted DNA fragments (B21-R and S7-R), and the *NPT* II sequence (*NTP* II-F and *NTP* II-R). The expression levels in the overexpression plants were detected according to the previously described method [40]. Specific primers for identification of knockout plants were designed based on the OsU3 and TaU3 promoter (OsU3p and TaU3p) [56]. Specific primers for identification of the mutation mode were designed outside of the dual sgRNA (s7-F, s7-R, b21-F, and b21-R). DNAMAN software (version 7.0.2) was used to analyze the hydrophilicity and hydrophobicity. TMHMM (TransMembrane prediction using Hidden Markov Models) software (http://www.cbs.dtu.dk/services/TMHMM/) was used to analyze the transmembrane domains.

### 4.5. Identification of the Function of LpABCB21 and LpABILS7 in SE

The scales of the overexpression lines and mutant lines were used as explants. The scales were cut into small pieces of approximately 0.5 cm^2^ and inoculated in the somatic embryo induction medium for dark culture. The wild-type plants were used as the control. The emergence time of somatic embryos was estimated. The results of somatic embryo induction were assessed after culture for 50 days.

## Figures and Tables

**Table 1:** The number of resistant calli under various transformation conditions.

Treatment	OE-*LpABCB21*	OE-*LpPILS7*	KO-*LpABCB21*	KO-*LpPILS7*
RC5.8	13/205 (6.34%)	21/247 (8.50%)	35/243 (14.40%)	31/225 (13.78%)
RC5.0	42/213 (19.72%)	46/231 (19.91%)	59/223 (26.46%)	67/246 (27.24%)

Note: Re-suspended solution and co-culture medium had pH 5.8 (RC5.8) or 5.0 (RC5.0). m/n (%), m represents the number of newly formed calli, n represents the number of inoculated calli, and % represents the percentage of newly formed calli in the inoculated calli.

**Table 2:** Comparison of genetic transformation efficiency before and after the improvement.

Method.	Type	Resistant Calli	Resistant Plants	Positive Plants	Germination Rate	Transformation Efficiency
Before	OE-*LpABCB21*	13	5	0	38.5%	0
	KO-*LpABCB21*	21	15	5	71.4%	33.3%
	OE-*LpPILS7*	35	7	1	20.0%	14.3%
	KO-*LpPILS7*	31	11	4	35.5%	36.4%
After	OE-*LpABCB21*	42	32	3	76.2%	9.4%
	KO-*LpABCB21*	46	41	19	89.1%	46.3%
	OE-*LpPILS7*	59	30	6	50.8%	20.0%
	KO-*LpPILS7*	67	52	24	77.6%	46.2%

## Supplementary Materials

Supplementary materials can be found at https://www.mdpi.com/1422-0067/21/18/6784/s1.

## Abbreviations

MS (Culture medium)	Murashige–Skoog
PIC (IAA analogue)	Picloram (Sigma–Aldrich, United States)
NAA (Auxin analogue)	1-Naphthaleneacetic acid (Sigma–Aldrich, United States)
6-BA (Cytokinin)	6-Benzylaminopurine (Tiangen, Beijing, China)
AS (Chemical material)	Acetosyringone (Tiangen, Beijing, China)
Cef (Antibiotic)	Cefotaxime (Tiangen, Beijing, China)
Hyg (Antibiotic)	Hygromycin (Sigma–Aldrich, United States)
Kan (Antibiotic)	Kanamycin (Tiangen, Beijing, China)
Rif (Antibiotic)	Rifampicin (Tiangen, Beijing, China)

## Appendix A

**Table A1:** Genetic transformation medium for Lilium pumilum DC. Fisch.

Media	Composition
Pre-cultivation/somatic embryo induction	MS (Murashige–Skoog) + 1.0 mg/L PIC (Picloram) + 0.2 mg/L NAA (1-Naphthaleneacetic acid) + 30 g/L Sucrose +7 g/L Agar; PH = 5.8
Re-suspension I	1/2 MS (NH_4_NO_3_-free) + 60 g/L Sucrose +100 µmol AS (Acetosyringone); pH = 5.8
Re-suspension II	1/2 MS (NH_4_NO_3_-free) + 60 g/L Sucrose +100 µmol AS; pH = 5.0
Co-cultivation I	MS (NH_4_NO_3_-free) + 1.0 mg/L PIC + 0.2 mg/L NAA + 100 µmol AS + 60 g/L Sucrose + 7 g/L Agar; PH = 5.8
Co-cultivation II	MS (NH_4_NO_3_-free) + 1.0 mg/L PIC + 0.2 mg/L NAA + 100 µmol AS+60 g/L Sucrose + 7 g/L Agar; pH = 5.0
Selection I	MS (NH_4_NO_3_-free) + 1.0 mg/L PIC + 0.2 mg/L NAA+30 g/L Sucrose + 7 g/L Agar + 400 mg/L Cef (Cefotaxime)+50 mg/L Kan (Kanamycin); pH = 5.8 (Overexpression)
	MS (NH_4_NO_3_-free) + 1.0 mg/L PIC + 0.2 mg/L NAA + 30 g/L Sucrose +7 g/L Agar + 400 mg/L Cef + 30 mg/L Hyg (Hygromycin); pH = 5.8 (Knockout)
Selection II	MS (NH_4_NO_3_-free) + 1.0 mg/L PIC + 0.2 mg/L NAA + 30 g/L Sucrose +7 g/L Agar + 400 mg/L Cef+25 mg/L Kan; pH = 5.8 (Overexpression)
	MS (NH_4_NO_3_-free) + 1.0 mg/L PIC + 0.2 mg/L NAA + 30 g/L Sucrose + 7 g/L Agar + 400 mg/L Cef + 15 mg/L Hyg; pH = 5.8 (Knockout)
Germination I	MS + 0.5 mg/L 6-BA (6-Benzylaminopurine) + 30 g/L Sucrose + 7 g/L Agar; pH = 5.8
Germination II	MS + 0.5 mg/L 6-BA + 0.88 g/L CaCl_2_ (calcium chloride) + 30 g/L Sucrose + 7 g/L Agar; pH = 5.8
