# Supplementary material for: Improvement of a Genetic Transformation System and Preliminary Study on the Function of LpABCB21 and LpPILS7 Based on Somatic Embryogenesis in Lilium pumilum DC. Fisch
Source: Int J Mol Sci. 2020 Sep 16;21(18):6784. doi: 10.3390/ijms21186784 (PMC7554901; doi:10.3390/ijms21186784)
Supplement: Supplementary file 1 [file ijms-21-06784-s001.pdf]

Supplementary Files

Improvement of a Genetic Transformation System and Preliminary Study on the Function of *LpABCB21* and *LpPILS7* Based on Somatic Embryogenesis in *Lilium pumilum* DC. Fisch.

Shengli Song [1], Rui Yan [1], Chunxia Wang [1], Jinxia Wang [1], Hongmei Sun [1,2]*

1 Key Laboratory of Protected Horticulture of Education Ministry and Liaoning Province, College of Horticulture, Shenyang Agricultural University, Shenyang 110866, China; ssl_syau@163.com (S.S.); yanrui2020@sina.cn (R.Y.); 2004500043@syau.edu.cn (C.W.); wangjinxia74@163.com (J.W.).
2 National and Local Joint Engineering Research Center of Northern Horticultural Facilities Design and Application Technology, Shenyang 110866, China
* Correspondence: sunhm@syau.edu.cn; hmbh@sina.com; Tel.: +8624-88487143

Supplementary R: The effects of $CaCl_2$ and light on the germination of somatic embryos.

As shown in Figure R1 and Table R1, non-transformed embryogenic calli (EC) were inoculated into various somatic embryo germination media for 50 days. The results showed that the germination of somatic embryos was more easily induced in the light culture than in the dark culture, and the coefficient of somatic embryo germination increased from 1.97 to 4.53. In the dark culture, addition of $CaCl_2$ (calcium chloride) to the original somatic embryo germination medium containing 0.44 g/L $CaCl_2$ to achieve the final concentration of 1.32 g/L increased the coefficient of somatic embryo germination from 1.97 to 5.23. In the light culture, when the somatic embryo germination medium contained 1.32 g/L $CaCl_2$, the coefficient of somatic embryo germination increased from 1.97 to 9.93. The germination efficiency of somatic embryos was the highest when non-transformed EC were inoculated in Germination II in the light culture (the light period was 16 h/8 h).

Table R1. The effects of $CaCl_2$ (calcium chloride) and light on EC germination.

Condition	$CaCl_2$（g/L）	No. of EC	No. of buds	Coefficient of EC germination
Dark culture	0.44	30	59	1.97
	1.32	30	157	5.23
Light culture	0.44	30	136	4.53
	1.32	30	298	9.93

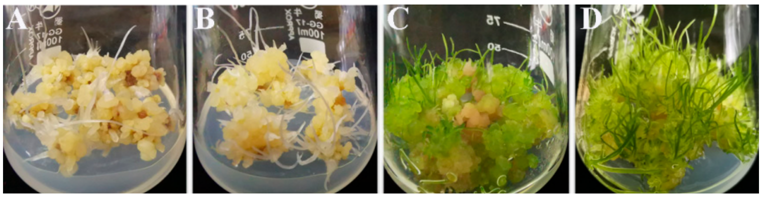

Figure R1. The effect of $CaCl_2$ (calcium chloride) and light on EC germination. A and C, EC were inoculated in germination medium containing 0.44 g/L $CaCl_2$, B and D, EC were inoculated in germination medium containing 1.32 g/L $CaCl_2$, A and B were cultured in the dark, C and D were cultured in the light (the light period was 16 h/8 h).

Table S1: The primers of overexpression and knockout vector construction

Primer name	Primer sequence（5'-3'）
LpABCB21-F	ACGCGTCGACCCGTAATGCTTGCCCTCTA
LpABCB21-R	TCCCCCGGGTGAAGAGGCTTTTGAGTGAAGTGCT
LpPILS7-F	ACGCGTCGACGAAAAACCTCTGCTTGGTGTTATTG
LpPILS7-R	TCCCCCGGGAATCATCTTCCATTCCCTCCCT
LpABCB21-BsF	ATATTATGGTCTCTGGCGTCTTGTTGGATGATCACTGGTT
LpABCB21-F0	GTCTTGTTGGATGATCACTGGTTTTAGAGCTAGAAATAGC
LpABCB21-R0	AACATGGCATCTTGGATGAGAACGCTTCTTGGTGCC
LpABCB21-BsR	ATTTATTGGTCTCTAAACATGGCATCTTGGATGAGAAC
LpPILS7-BsF	ATATTATGGTCTCTGGCGGTGTTGATATTAGTCCCTGGTT
LpPILS7-F0	GGTGTTGATATTAGTCCCTGGTTTTAGAGCTAGAAATAGC
LpPILS7-R0	AACTTGTCGTCTATCTTGACTACGCTTCTTGGTGCC
LpPILS7-BsR	ATTTATTGGTCTCTAAACTTGTCGTCTATCTTGACTAC

Note: The underlined parts are the restriction enzyme cutting sites and the protective base, and the red letters are the sgRNA sequences.

Table S2: The primers for identification of transgenic plants

Prime name	Primer sequence（5′-3′）
NTP II-F	GATTGAACAAGATGGATTGCACG
NTP II-R	TCATTTCGAACCCCAGAGTCC
35S-F	GGACCTAACAGAACTCGCCGT
S7-R	TCCGATCAAAAAGATAATCCCG
B21-R	AACCCTCCGAAAAGAACCGT
OsU3p	GCCCATTACGCAATTGGACGACAAC
TaU3p	CTCACAAATTATCAGCACGCTAGTC
s7-F	TCGGAGGAAGCCTTGGATGG
s7-R	CCATTGGACTTGGTGCTGGATAAC
b21-F	GTCTGCCACTCCTAGCAATACGG
b21-R	CCAAGACCCAATCCTACGGC

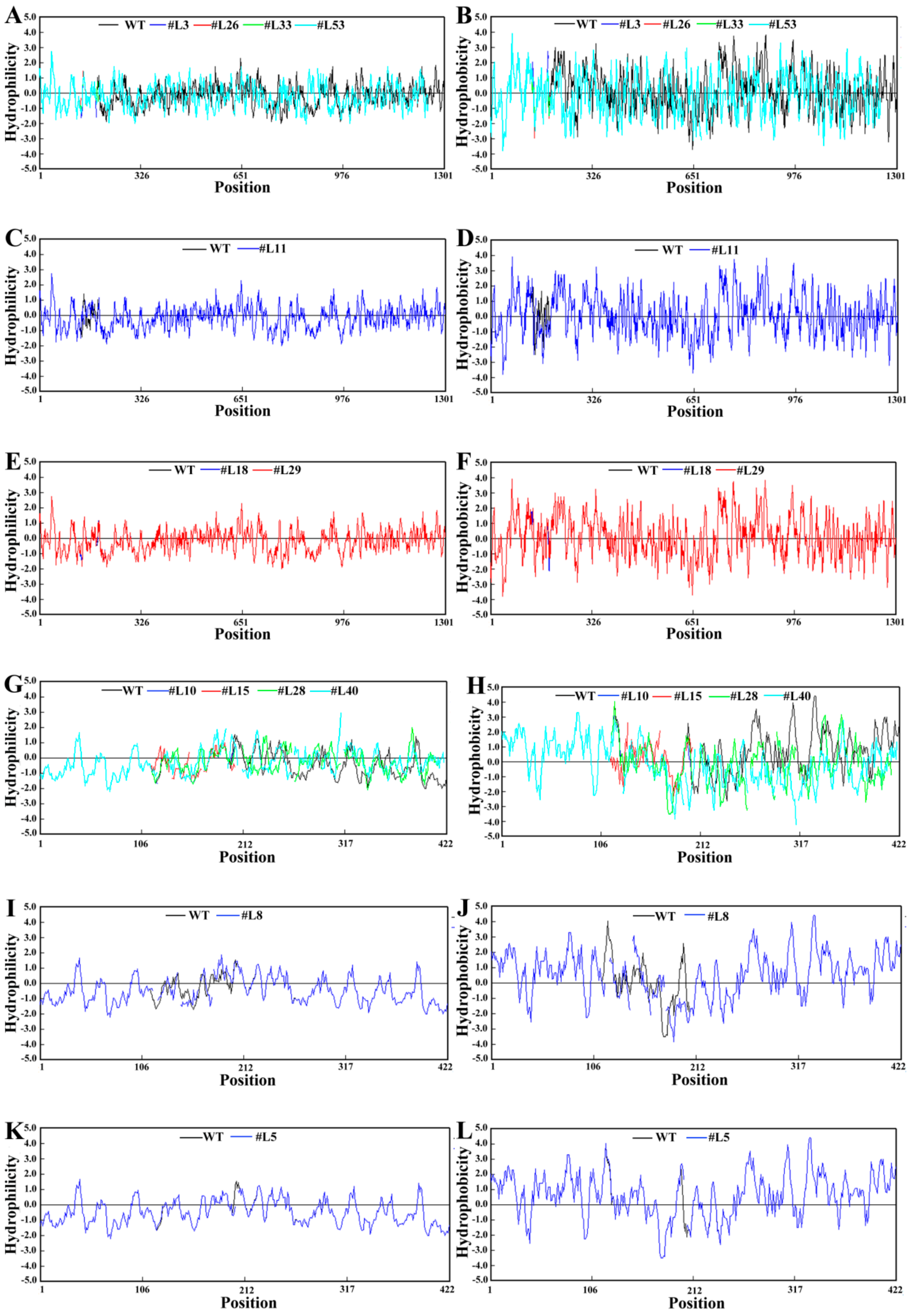

Figure S1: Analysis of the hydrophilicity and hydrophobicity of *LpABCB21* (A-F) and *LpPILS7* (G-L) mutant lines

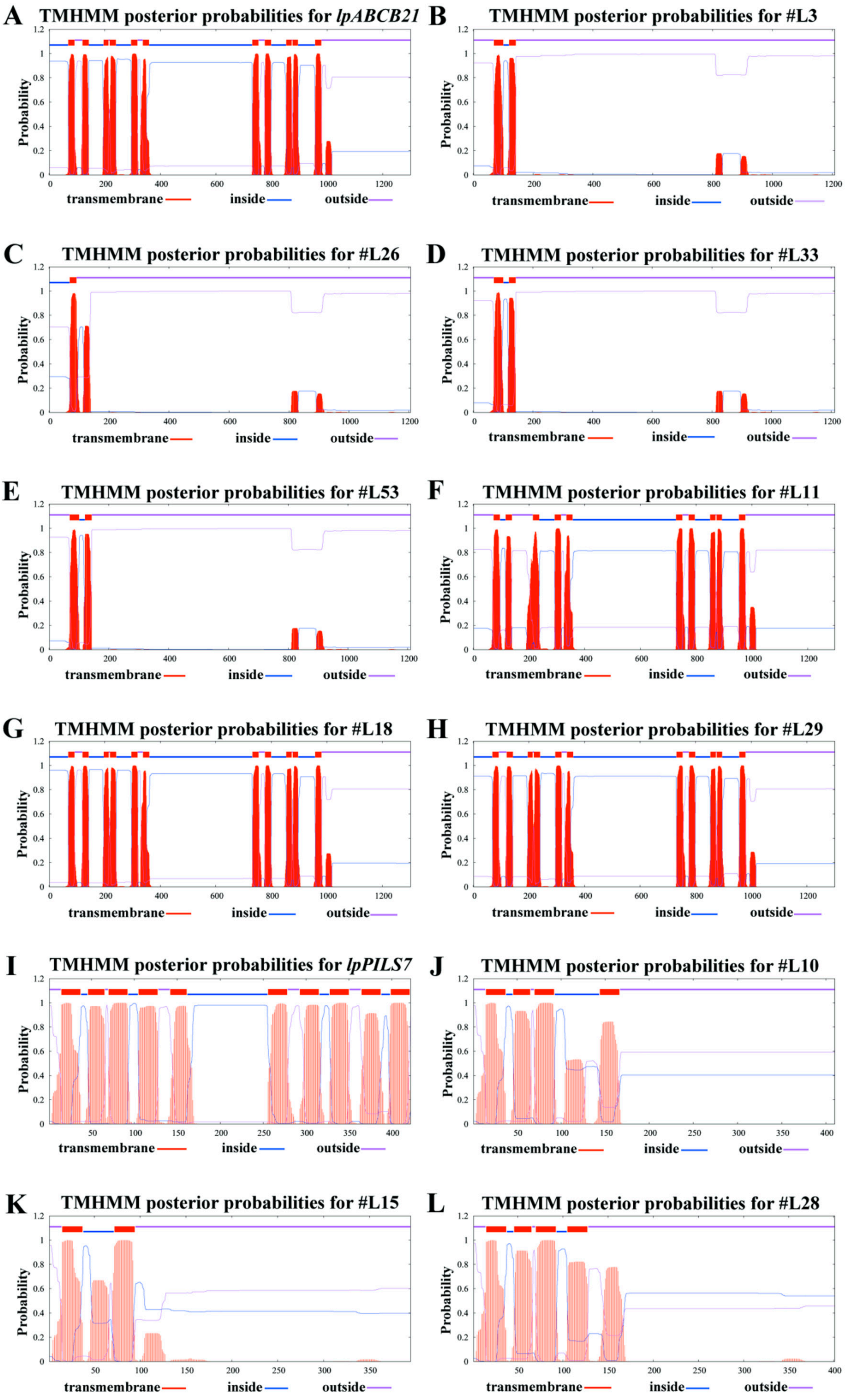
A TMHMM posterior probabilities for *lpABCB21*
B TMHMM posterior probabilities for #L3
C TMHMM posterior probabilities for #L26
D TMHMM posterior probabilities for #L33
E TMHMM posterior probabilities for #L53
F TMHMM posterior probabilities for #L11
G TMHMM posterior probabilities for #L18
H TMHMM posterior probabilities for #L29
I TMHMM posterior probabilities for *lpPILS7*
J TMHMM posterior probabilities for #L10
K TMHMM posterior probabilities for #L15
L TMHMM posterior probabilities for #L28
Probability
transmembrane
inside
outside

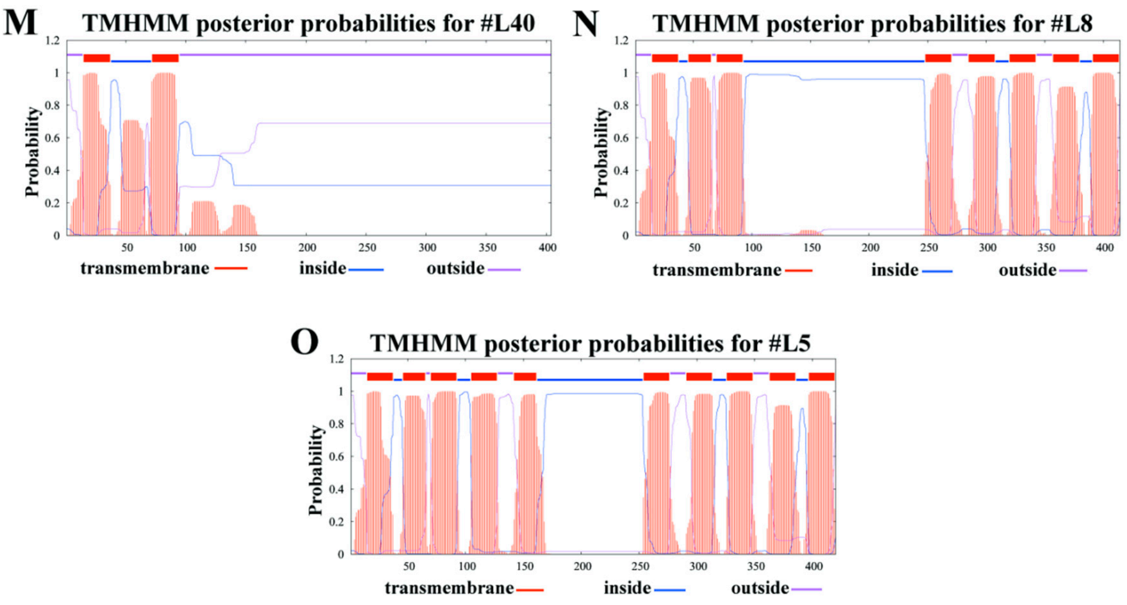

Figure S2: The transmembrane domain analysis of *LpABCB21* and *LpPILS7* mutant lines. A was the predicted transmembrane domain of wild-type *LpABCB21*, I was the predicted transmembrane domain of wild-type *LpPILS7*, B-H were the predicted transmembrane domain of *LpABCB21* mutant lines, and J-O were the predicted transmembrane domain of *LpPILS7* mutant lines.

Sequence information

Note: The bold characters with borders represent the start and stop codons. The bold characters with yellow shade represent the sgRNA sequence. The red characters represent the NGG domains.

>*LpABCB21* 4086bp

CCGTAATGCTTGCCCTCTAAATTGCAACAAAGCACGTCTCTAAGTCCTTCAAGTGCTACCATTTCAGATTCAGG
ACTCAGCAATCATAGTTGTGGGATCATTCTACCTAGTTCTTGCATGGTACTAACTGAACTATAGAAACGGAGAT
TTAAGTACGATAACTCTTGTAAGCTAGATATAACA**ATG**GGGAGAGAGAAGGAACCTGTGAGCTCTTCGATAGCT
CTGATGCCCATGGAGGACCAAATCAATGGCCATTCTGATGTGAATTCGTCTGCCACTCCTAGCAATACGGTGGT
AGAGGAAAGGGAGAAGAAGACAGAGAGCCAGGACGATCCCAAATACACGATACCATTCTATAAGTTGTTCTCAT
TTGCGGATTCCACCGATATCATCTTGATGATTGTTGGTACATTTGGAGCTATAGGAAATGGAGCTGCGCTGCCT
CTGATGACGGTTCTTTTCGGAGGGTTGATGAATTCCTTTGGAGAAGCTGCCGATAAAAACGATGTAGTTCGAAG
AGTTTCGAAGGTAGCGCTTGAATTTGTGTATCTGGCGATAGGATCGGGCGTGGCATCCTTTCTTCAGGT**GTCTT**
GTTGGATGATCACTGGGGAGAGGCAATCAGCACGAATAAGAAATTTATACTTGAAAACGATATTACATCAAGAA
GTCGCATTTTTCGACATGGAAACAAACACGGGAGAAGTTGTGGGGAGGATGTCTGGTGACACT**GTTCTCATCCA**
AGATGCCATGGGTGAAAAGGTTGGGAAGTTCATTCAACTAATGTCATCGTTCTTTGGGGGATTTATAGTAGCAT
TTGCTCAAGGTTGGCTTCTTACCTTCGTTATGATCACTTCGATTCCTCTGCTTGTGGCCGCGGGCGCAGCAATG
TCGATCATTATAGCCAAAACGGCATCGCGCGGACAAGCAGCTTATGCAGAGGCAGCAGTTGTTGTAGAACAGGC
GATAGGGTCAATCAGAACAGTTGCATCTTTTACGGGAGAAAAGCAATCCATTGATAGGTATAATAAGTCGCTTA
CAGGCGCATACAGTTCTAGTGTTCAAGAGGGTCTGGCCGTAGGATTGGGTCTTGGTACCGTCTCAATGGTGATG
TTCCTGGGTTATTCCTTGGGCATTTGGTATGGATCAAAGTTGATATTAGATAAAGGATACACTGGTGCCAGCGT
CATGAATGTGATGTTTGCTGTCATTACTGGATCCGTTTCTTTAGGCCAGACATCTCCATGCATGACCGCATTTG
CTGCAGGACAAGCTGCAGCCTTCAAGATGTTTGAGATAATCAATAGAATACCAGAGATAGATGCTTATGACATC
AGGGGAAAGATAATGGAGGGAATTAACGGACATATAGAATTTAGAAATGTCTCTTTCAGTTATCCAGCCAGACC
AGATGAGCAAATATTCAATGGATTCTCTCTATTCATTCATAGTGGAAAGACAGCGGCATTAGTTGGACAGAGTG
GAAGTGGAAAATCAACGGTCATCAGTCTGATAGAACGGTTCTATGATCCGCAGGCTGGTGAAGTTCTCATTGAT
GGCATAAATATCAAGGAGTTTCAATTGAGATGGCTAAGGGGAAAAATTGGACTTGTCAGTCAGGAACCAATCTT
ATTTGCTTGTAGCATCAGAGATAATATCGCCTATGGCAAGGATGGTGCAACTACAGAAGAAATCAGAGCTGCGA
CAGAGCTTGCAAACGCTGCAAAATTTATAGACAAAATGCCTCAGGGTATTGATACCATGGTCGGTGAGCATGGA

ACTCAGCTATCAGGTGGGCAGAAGCAGAGAATTGCCATTGCAAGAACAATTCTAAAAGACCCGCGAATTTTACT
ACTAGATGAAGCCACTAGTGCCCTGGATGCAGAATCTGAAAGAATTGTACAGGAGGCGCTTGATCGTGTGATGA
CAAATCGGACCACTGTAATAGTTGCTCATCGCCTGACCACCGTTAGAAATGCAGATTCGATCTCTGTTATACAT
CGGGGATCAATCGTTGAGCAAGGTGCACACGAAGAGCTACTAAAGAATCCAAATGGGGCTTACTTCCAACTCGT
ACAATTGCAGGACATGAATCGGAAATCAGACCATGCACCGCGACTTGACCAGGATAAACGTGATACTTCATTTA
ATCTAGGAAGAAGTGCAAGCCAACATCAGTCATTCAAGCGTTCTTTCAGTCGAGGAGCATCGATCGGAGGTAGC
GGACGCCGATCATTCTCAGCACCATTTGGTTTTCTTATAGGAATGAACATCCAAGGAAACAGATTGGAGGAACA
AGACACTGAAGTTCCTTCAGAAGAACCAGAAAATGTTCAAGTCCCCCTTAGACGCCTCGCTTCTCTTAACAAGC
CAGAAATCCCAGTGTTGATTATCGGAGCAGTTGCTGCGATAGTGAATGGACTAATATTTCCAGTATTTGGAATT
CTGCTCTCTGGTGTGATAAGTACATTTTACAAGCCACCAGCACAGCTTCGGAAGGATTCCAACTTTTGGGCAGT
GATTTTCCTGGTATTTGCAGCGGTTTCTTTTATAGCACCTCCAGCTCGATCGTATTTCTTTGCGATTGCTGGAT
CCAAGTTGATTAGACGAATTCGAGCGATGGCATTTGAGAAGGTGATTCACATGGAGATAGGTTGGTTTGATGAA
TCTGAGAATTCAAGTGGGGCAATTGGAGCCAGACTATCGGCTGATGCAGCAACAGTTAGGAGTCTTATGGGTGA
TGCGCTCGCACTAATAGTTCAGAACATCGCCACTTTAGCAGCTGGTTTGGTGATCGCTTTTACCGCCAATTGGC
AACTATCTCTGATTATCTTGGCTTTGATACCTGTCATGGGTTTAAATGGATGGATCCAGATGAAGTTCATGACG
GGATTCAGTGCAGATGCAAAGATGATGTATGAGGAAGCAAGTCAAGTTGCCAACGATGCAGTTGGAAGTATAAG
AACGGTTGCATCCTTCTCAGCTGAAGAGAAGGTGATGGAACTATACAAAAAGAAATGTGAAGGTCCTATGAGAA
CTGGAATTAGGCAAGGACTGATCAGTGGTATCGGTTTTGGTATCTCCTTTCTCTTGCTATTTTGTGTCTATGCA
ACCAGTTTTTATGCTGGAGCTCGGCTTGTTCAGGCTGGAAAGACCACTTTTGACAAAGTTTTCCGGGTTTTCTT
TGCTCTTTCCATGGCAGCTGTTGGAATTTCTCAATCTAGCTCCCTAGCACCAGATTCAACCAAAGCCAGATCTG
CTACAGCTTCTATATTTGCTATTCTCGACAGGAAATCTAAGATAGATTCGAGTGATGGTTCTGGAATAACCTTA
GATGTATTGAAGGGGAATATTGAGTTTCGCCATGTCAGTTTCAAATATCCTACAAGGCCAGACGTGCAGATTTT
CCAAGATTTGTGCTTGGCAATACAATCAGGGAAGACTGTTGCACTAGTTGGTGAGAGTGGGAGTGGAAAATCGA
CGGCAGTATCATTGTTGCAGCGATTTTACGACCCTGATTCAGGTCAAATATTGATAGATGGAACTGAAATACGA
AAATTCCAGTTGAGATGGTTGAGGAAGCAGATGGGTCTGGTGAGTCAGGAGCCAGCTTTGTTCAATGATACAAT
CCGAGCTAATATTGCATATGGAAAGGAAGAGCAAGCCAGCGAGTCTGAGATTATAACTGCCTCGGAATCGGCGA
ATGCACACAAATTTATTAGTACTTTGCAGCAGGGGTATCAAACAGTAGTCGGGGAGCGAGGGATCCAGTTATCC
GGTGGACAGAAGCAGCGGGTGGCTATTGCACGTGCCATAGTGAAGGATCCTAAAATCTTGTTACTTGATGAAGC
TACTAGTGCACTTGATGCTGAATCTGAACGTGTAGTTCAAGATGCATTAGACCGTGTGATGGTAAACCGAACCA
CGGTGATCGTTGCTCACAGACTGTCAACAATAAAAGGGGCTGATTTAATTGCGGTAGTTAAAAATGGTGTGATC
AAAGAAAAGGGGAGGCATGAGACCTTGCTAAAAATCAAGGATGGAGTTTATGCTTCCTTAGTAGCACTTCACTC
AAAAGCCTCTTCA**TGA**

>*LpPILS7* 1461bp

GAAAAACCTCTGCTTGGTGTTATTGAGCTTGGGCTAAGCATACATAACACATTTGAAACACATTGAAAGGTTGA
AATCTCGAGCTAATCTCTAGCAAACAAAGCAGCAAAG**ATG**GGGTTTTTGTCTCTGTTCGAGGTGGCGACAATGC
CAGTCTTGCAGTTCATGCTCATTGGCATGCTAGGAGCCTTTCTGGCAACCGATTATAGCAACATTCTATCGGCC
AACGCCCGGAGAGACATCAACAAGTTGGCATTCTATGTGTTCTCACCATCAATAGTATTTTCAAGCCTAGCAAA
AGCGGTAACGCTTAAAGACCTCATCTCTTGGTGGTTTATGCCGGTTAACATCGGGATTATCTTTTTGATCGGAG
GAAGCCTTGGATGGATAGCAGTGAAGATCTTGAGACCAGAGCGCCACCTTGAAGGGGTTGTCATTGGCAGCTGC
TCAGCAGCCAACTTGGGCAATCTG**GTGTTGATATTAGTCCCTG**CGGTCTGCAATGAAGCAGCCAGTCCGTTCGG
TGATTCAGCTTTATGTCGTGCCAGAGGGCTTTCCTATGTATCAATGTCTATGTCGCTTGGGAATATCTTTATAT
GGACCCATACCTACAGTCTGATGCGGAAATCAAGTATTTTGTACAACCAAAAGCATAACCAGAACCAACACGAG
ATAAGCAACACAGACTCGGAAGTGAGTAGCGAAGCTATT**GTAGTCAAGATAGACGACAA**AGGAAGCTCTAGTGA
TCAGGAGGTATTGGTCCAGTCCACAATTACATCTACAGATGCTTCAACAGATGAAAAGACGATACCGCTGTTAT
CCAGCACCAAGTCCAATGGCAACAAGTTGAGTTTTCAGGACAGATTGAAAGGGCTTAACAAAGTACTAGAGGAA
TTATTTGCACCCCCTACAATTGCTGCGATAATCGGCTTAATTGTTGGCGCGATCCCTTGGCTGAAATCACTTAT
CATCGGATCGGACGCCCCTCTTAGAGTAATTGAAGATTCTATCACGTCACTCGGAAATGGAATGCTGCCATGCA
TTATCCTCATTTTAGGAGGAAACCTAACTCAAGGTCTACGAAAATCCACGATAAAGCCTTCACTGATTGTAGTG
ATCATCATCGTCCGATACCTATTTCTTCCTATTGCTGGGATTGCTGTAGTGAAAGCAGCAGGTGAACTTGGATT
CCTGGCTGAAGATTCTTTGTATCGCTATGTGTTAATGATCCAGTTCACTCTCCCACCAGCCATGAGTATTGGTA
CAATGGCTCAACTTTTCGATGTTGCGAAGGAAGAGTGCTCGGTCATATTCCTATGGACATACTTGGCAGCTCTG
GTAGCAATTTCGATATGGCCGACTGTCTTCATGTGGATCTTGACC**TAG**AATACCTTGGTACATCAAGCAAGTGT
TAATATTCTTTATCTGGTGTTGCAAGATTTTGGAGGGAGGGAATGGAAGATGATT
